# Management of ischaemic stroke in the acute setting: review of the current status

**DOI:** 10.5830/CVJA-2013-001

**Published:** 2013-04

**Authors:** Kalpesh Jivan, Kaushik Ranchod, Girish Modi

**Affiliations:** Division of Neurology, Department of Neurosciences, School of Clinical Medicine, Faculty of Health Sciences, University of the Witwatersrand, Johannesburg, South Africa; Division of Neurology, Department of Neurosciences, School of Clinical Medicine, Faculty of Health Sciences, University of the Witwatersrand, Johannesburg, South Africa; Division of Neurology, Department of Neurosciences, School of Clinical Medicine, Faculty of Health Sciences, University of the Witwatersrand, Johannesburg, South Africa

**Keywords:** stroke, intravenous r-tPA, recanalisation treatments

## Abstract

**Abstract:**

Acute ischaemic stroke can be treated by clot busting and clot removal. Thrombolysis using intravenous recombinant-tissue plasminogen activator (IV r-TPA) is the current gold standard for the treatment of acute ischaemic stroke (AIS). The main failure of this type of treatment is the short time interval from stroke onset within which it has to be used for any benefit. The evidence is that IV r-TPA has to be used within 4.5 hours.

Other modalities of treatment are not as effective and need more scrutiny and examination. The available modalities are intra-arterial thrombolysis and clot-retrieval devices. Not unexpectedly, recanalisation treatments have flourished at a rapid rate. Although vessel recanalisation is vital to increasing the possibility of significant tissue reperfusion, clinical trials need to emphasise functional outcomes rather than reperfusion/recanalisation rates to adequately assess success of these devices/techniques.

Our view is that until these treatments become proven in large-scale studies, a greater endeavour should be made in resource-limited settings to expand facilities to enable intravenous r-tPA treatment within the 4.5-hour period following onset of stroke. The resources required are small with the main costs being a CT scan of the brain and the cost of r-tPA. This can easily be done in any emergency facility in any part of the world. What is needed is public awareness, and campaigns of ‘stroke attack’ should be revisited, especially in the resource-limited context. This approach at present will halt to some extent the stroke pandemic that we are facing.

## Abstract

Stroke is the third leading cause of death worldwide, resulting in approximately 5.7 million deaths annually.^1^ With current treatment options, this number is projected to rise to 6.5 million in 2015 and to 7.8 million in 2030. The global focus on stroke treatment is based on these figures and reflects the impact stroke has on society. In a recent review, taking into account 120 cost studies in developed countries, the average costs of stroke ranged from $468 to $146 149.^2^ There is limited information regarding the cost of stroke in developing countries. The approximate cost of ischaemic stroke in Togo is EUR428.80.^3^ Currently there is inadequate information regarding the cost of stroke in South Africa.^4^

The main issue with regard to stroke is outcome. Clinical outcomes of stroke have been reviewed in 174 acute stroke trials.^5^ Death occurred in 76% of patients in the trials, impairment of body function and structure in 76%, disability (activity limitations) in 42%, and adverse social impact or restricted quality of life occurred in only 2% of patients. Functional outcomes are the main cause of stroke cost. Stroke is the eighth most significant cause of life lost due to illness and the ninth most important cause of disability in South Africa.^4^

The ultimate goal in stroke management is to reverse the stroke and leave no disability. This has however not been possible to date. From a pathophysiological point of view, in the acute setting, this could be achieved by improving perfusion in the ischaemic area.

Current models of stroke pathology indicate the area of infarction following a stroke is surrounded by an ischaemic penumbra. Cerebral blood flow (CBF) of below 10–12 ml/100 g/min results in irreversible neuronal injury/infarction.^6^ Within an hour of hypoxic ischaemic insult, this core of infarction is surrounded by an oligaemic zone called the ischaemic penumbra where autoregulation is ineffective. The penumbra phase generally begins when CBF flow falls below 20 ml/100 g/min.^6^ Cellular integrity and function are preserved in this potentially salvageable penumbra for variable periods of time. Although little can be done to save the infarcted core, it is the penumbra that is the target of salvage therapies.

## Methods

A PUBMED search was conducted using the keywords ‘acute stroke management’, ‘interventional devices for acute stroke’, ‘intravenous thrombolysis’, ‘intra-arterial thrombolysis’, ‘guidelines for stroke management’ and ‘prevention of strokes’ from 1995 to 2012.

## Results

Three types of treatment in the acute setting have emerged to salvage the penumbra, reduce the area of infarction and improve stroke outcome. These include clot busting, clot removal and prevention of recurrence of stroke.

## Clot busting

This is achieved by intravenous or intra-arterial thrombolysis.

## Intravenous recombinant tissue plasminogen activator (IV r-tPA)

IV r-tPA when used within three hours of stroke onset in select patients with acute ischaemic stroke (AIS) is the only US Food and Drug Administration (FDA)-approved thrombolytic treatment for stroke.^7^ South African guidelines recommend it should be administered at a hospital where rapid triage of stroke patients is possible, with established protocols for the use of r-tPA and where good post-treatment care is available.^4^

In 1995 the landmark NINDS trial randomised 624 patients with AIS to receive 0.9 mg/kg of IV r-tPA or placebo within three hours of stroke onset.^8^ Three months after treatment, 50% of patients in the treatment arm had minimal or no disability compared to 38% of patients in the placebo study arm: a 12% absolute improvement. Although 6.4% of patients treated with IV r-tPA developed symptomatic intracerebral haemorrhage (sICH) compared to 0.6% of patients given placebo, the death rate in the two treatment groups was similar at three months (17 vs 20%). This was the first time a treatment for stroke had improved or reduced disability significantly.

In two other large, randomised, double-blinded phase 3 trials, the European Cooperative Acute Stroke study (ECASS) and ECASS-II, IV r-tPA was not more effective than placebo in improving neurological outcomes 90 days after stroke.^7^,^9^ A dose of 1.1 mg/kg of IV r-tPA was used in ECASS and a dose of 0.9 mg/kg was used in ECASS II. In both trials, patients were treated up to six hours after stroke. The ATLANTIS A and ATLANTIS B trials from North America, both placebo-controlled, doubleblinded, randomised trials, also did not support the use of IV r-tPA beyond three hours of ischaemic stroke onset.^10^,^11^

Taking into account the findings of all these studies, the 2007 American Heart Association/American Stroke Association (AHA) guidelines recommended the dosing regimen of 0.9 mg/kg (maximum 90 mg) for selected patients who may be treated within three hours of onset of AIS (class I, level of evidence A).^12^ Ten per cent of the dose is given as an initial intravenous bolus and the rest is infused over one hour provided there are no contraindications for the treatment.12 However, this dose is not universally accepted, with most Japanese studies continuing to support the use of 0.6 mg/kg.^13^

Treatment benefit is time dependent and the number needed to treat (NNT) to get one more favourable outcome drops from four during the first 90 minutes to seven at three hours, and towards 14 between three and 4.5 hours.^14^,^15^ This implies that there is no potential benefit beyond three hours.

In 2008 the ECASS III trial showed that IV r-tPA administered within three to 4.5 hours of stroke onset may offer a moderate benefit when applied to all patients with potentially disabling deficits.^15^ The incidence of intracranial haemorrhage was higher with IV r-tPA than with placebo in this study [27.0 vs 17.6% for any intracranial haemorrhage (*p* = 0.001) and 2.4 vs 0.2% for symptomatic intracranial hemorrhage (*p* = 0.008)], but mortality did not differ significantly between the two groups.

In the joint-outcome table analysis of the ECASS III trial, the number needed to treat to benefit (NNTB) was 6.1 and the number needed to treat to harm (NNTH) was 37.5, which indicates that for every 100 patients treated in the three- to 4.5-hour window, 16.4 will have a better outcome and 2.7 will have a worse outcome by ≥ 1 level on the modified Rankin score (mRS) of global disability.^16^ In other words, among individuals matching the ECASS III cohort, as a result of treatment with IV rTPA in the three- to 4.5-hour window, approximately one in six patients have a better outcome and one in 35 have a worse outcome.

Furthermore, in a study determining the NNTH following IV r-tPA, most patients who experienced sICH after IV r-tPA therapy had severe baseline insults and were destined for a poor outcome.^17^ In other words, the sICH may have caused temporary early worsening, but is unlikely to have altered the final functional outcome. Using a 15-variable prognostic model derived from the placebo group in NINDS 1 and 2, it was found that the NNTH ranged from 29.7 to 40.1.^17^ This implies that among individuals matching the NINDS cohort, for every 100 patients treated with IV r-tPA , only one will experience a severely disabled or fatal final outcome.

Based on these findings, the AHA issued revised guidelines that expanded the window for IV r-tPA treatment from three to 4.5 hours in eligible patients.^18^ In a recent phase 2B trial comparing the use of alteplase to tenecteplase in eligible patients within six hours of onset of acute ischaemic stroke, tenecteplace was shown to be superior in terms of significantly better clinical improvement and reperfusion.^19^ A phase 3 trial is needed to confirm this.

There is no evidence showing benefit with any other intravenously administered thrombolytic agents, including streptokinase, reteplase, urokinase, anistreplase and staphylokinase for use in acute stroke. These should be avoided in routine clinical practice outside the context of a clinical trial.^4^ Studies with desmoteplase and ancrod, a defibrogenating enzyme derived from snake venom, are underway.

Risks and benefits of treatment with IV r-tPA for AIS should be discussed with the patient or family before administration.^12^ Written consent has been deemed as not necessary by health authorities.^12^ Adverse events of IV r-tPA include intracranial haemorrhage, anaphylactic reaction, angio-oedema and myocardial rupture.^12^ In a recent international, multicentre, randomised, open-treatment trial, assessing the benefits and harms of IV r-tPA given within six hours of AIS, deterioration due to swelling of the infarct and sICH in the first seven days was more significant in those patients who received IV r-tPA compared to those who did not.^20^ Patients receiving IV r-tPA also had significantly more non-fatal extracranial haemorrhages.^20^

Difficulties in administering IV r-tPA within 4.5 hours include early recognition of signs of stroke by the patient or family members, early evaluation of the patient by paramedics, rapid transport of patients to stroke centres, availability of radiological services, and appropriate evaluation by an experienced clinician as to the suitability of thrombolytic therapy. Indications and contraindications for IV r-TPA are described in Table 1.

**Table 1:** Use Of IV Thrombolysis In AIS

**Indications for IV thrombolysis in AIS**
• Age > 18 and < 80 years.^15^
• Symptoms onset within 4.5 hours.^15^
• Measurable deficit on the National Institutes of Health Stroke Scale (NIHSS) examination.^4^
• CT scan does not show haemorrhage or non-stroke cause of deficit.^4^
**Contraindications for IV thrombolysis in AIS**
• Rapidly improving or minor symptoms.^12^
• Stroke within past three months.^12^
• Previous intracerebral haemorrhage or vascular malformation.^12^
• Patient has symptoms suggestive of subarachnoid hemorrhage.^12^
• Bleeding diathesis.^15^
• Arterial puncture at non-compressible site or lumbar puncture within the last seven days.^12^
• Gastrointestinal or urinary tract bleeding in the last 21 days.^12^
• Invasive surgery or delivery in last two weeks.^12^
• Platelet count < 100 000 /µl.^12^
• On treatment with heparin with prolonged aPTT.^12^
• On treatment with oral anticoagulation with INR > 1.7.^12^
• Serious head trauma within the previous three months.^15^
• Systolic blood pressure > 185 mmHg, diastolic blood pressure > 110 mmHg.^4^
• No myocardial infarction in the previous three months.^12^
• Seizures at onset of stroke with postictal residual neurological impairments.^12^
• Blood glucose < 50 mg/dl (2.7 mmol/l).^4^,^15^
• Multilobar infarction (hypodensity > 1/3 of cerebral hemisphere).^12^

## Intra-arterial (IA) thrombolysis

AHA guidelines recommend that IA thrombolysis can be considered an option for treatment of AIS due to occlusions of the MCA only if given within six hours of stroke onset in patients who are not otherwise candidates for IV r-tPA.^12^ This would include patients described in Table 1.

Prolyse in Acute Cerebral Thromboembolism (PROACT) II, the only randomised study that has examined the safety and efficacy of IA thrombolysis in patients with AIS, compared outcomes of 121 patients in the treatment group treated with intra-arterial recombinant prourokinase (r-proUK) and heparin within six hours of stroke onset, with outcomes of 59 patients in the control group treated with heparin alone.^21^ In this study, the technique involved placing an infusion microcatheter under angiographic guidance into a proximally (M1 or M2 segment of the middle cerebral artery) occluding thrombus; 4.5 mg of r-proUK was infused at a rate of 30 ml/h into the thrombus. One hour later, a second angiogram was done through the microcatheter and the remaining 4.5 mg of r-proUK was infused over the next hour. Another diagnostic carotid angiogram was performed at two hours to assess final vessel patency. Theoretically, IA thrombolysis may offer a higher dose of thrombolytic drug delivery to the clot with fewer systemic complications and higher recanalisation rates.^22^

In the PROACT II treatment group, the recanalisation rate was 66% with 40% of patients reaching functional independence within 90 days.^21^ In the control group, the recanalisation rate was 18% with 25% of patients reaching functional independence. sICH occurred in 10% of treated patients compared to 2% of controls, but the increase in sICH did not affect mortality rates, which were 25 and 27%, respectively.

These results cannot be compared to any of the IV r-TPA trials as the study methods and patient selections were different. Also, one has to factor in the need for greater skill and the risks of catheterisation when doing IA thrombolysis. There is no randomised controlled study comparing IV r-TPA versus IA r-TPA with/without heparin.

In a recent meta-analysis, the outcomes of IV r-tPA from three randomised, controlled trials, namely, Middle Cerebral Artery Embolism Local Fibrinolytic Intervention Trial (MELT), PROACT and PROACT II were pooled together and statistically averaged using odds ratios for 130 patients. This was then compared to the outcomes of IA thrombolysis by sensitivity analysis.^23^ Using these statistical methods, no benefit was shown from IA thrombolysis.^23^

However the obvious criticism of this meta-analysis is that data was obtained from three different trials in which the times to treatment were not uniform. In the PROACT and PROACT II studies, the authors could not obtain information of arrival time to hospital and treatment with IV r-tPA.

Therefore while there is a suggestion in the literature that IA thrombolysis is not significantly better than IV r-tPA, robust studies using these two arms are still lacking.^23^ Despite this the results of this IA r-TPA trial were promising. Unfortunately, treatment with prourokinase + heparin was not approved by the FDA, which demanded a confirmatory study that was never performed.^24^ The availability of intra-arterial thrombolysis should generally not preclude the intravenous administration of r-tPA in otherwise eligible patients.^12^

## Clot removal

## Mechanical clot retrieval

Mechanical revascularisation for acute stroke may be considered in large-vessel occlusions. Recently, complex devices have been developed. These can be divided into two major groups:

• Proximal devices, which apply force to the proximal base of the thrombus.
• Distal devices, which approach the thrombus proximally but a guide wire and microcatheter is advanced distally and then unsheathed to apply force to the distal base of the thrombus. This group includes snare-like, basket-like or coil-like devices.

Mechanical thrombectomy can also be performed in patients who have received IV r-tPA and in those who are not candidates for IV r-tPA. Results of multiple non-randomised trials have shown these devices to be safe for clot removal in acute ischaemic stroke patients presenting up to eight hours from onset of the event.^22^ It requires the knowledge and skills of trained neuro-interventionalists with experience in the use of the devices. Two FDA-approved devices are available specifically for mechanical thrombectomy: the MERCI retriever and the penumbra system.

The Mechanical Embolus Removal in Cerebral Ischaemia (MERCI) retriever is a cork-screw-shaped device consisting of a flexible nitinol wire in five helical loops. It allows for placement distally and then *en bloc* removal of the thrombus. The MERCI and Multi MERCI trials evaluated the safety and efficacy in the setting of stroke within eight hours of onset.^25^,^26^

The MERCI trial included 151 patients with anterior or posterior circulation stroke secondary to large-vessel occlusion.^25^ Primary outcomes were recanalisation defined as a thrombolysis in myocardial infarction (TIMI) score of 2–3 and safety.^25^ Secondary outcomes were modified Rankin score (mRS) and NIHSS scores at 30 and 90 days and death, myocardial infarction or second stroke within 30 days. A good neurological outcome was defined as MRS < 2 (i.e. either asymptomatic or no significant disability) or a NIHSS score improvement ≥ 10 points.

Recanalisation (TIMI 2–3) was achieved in 46% of treated patients, with these patients having better neurological outcomes. The risk of stroke, MI or death at 30 days was 40% and mortality rates at 90 days were 43.5%. Increasing age and a higher admission NIHSS score were associated with higher mortality rates. Clinically significant procedural complications occurred in 7.1% of patients and sICH in 7.8% of patients.

In the Multi MERCI trial, 160 patients were treated within eight hours of stroke onset.^26^ In this study, prior treatment with IV r-tPA, mechanical clot disruption, IA thrombolysis and other adjunctive therapies were allowed. IV r-tPA had been administered to 29% of the participants without recanalisation prior to the procedure. Recanalisation was achieved in 55% of patients with the retriever alone and in up to 68% when adjunctive therapies were used. At 90 days good neurological outcomes (mRS ≤ 2) were achieved in 36% of patients and NIHSS scores improved > 10 points in 26% of patients.

Given that clot burden in the internal carotid artery terminus and basilar artery can be substantially higher and therefore less likely to be recanalised with thrombolytics, the data suggest that the device provides an advantage over IA thrombolytic therapy alone for all large-vessel occlusions.^26^ Treatment with IV r-tPA prior to MERCI device deployment did not increase the chance of sICH. Overall mortality at 90 days was 34%. Although the mortality rates were relatively high in both MERCI and Multi MERCI trials this most likely represents the overall stroke severity of the patients enrolled.

The penumbra system works proximally to disrupt and aspirate the thrombus. It comprises a series of devices, primarily an aspiration catheter, with a distal wire to keep the catheter clear, and a grasping device designed to remove harder thrombus if the aspiration device fails to recanalise the vessel.

A prospective multi-centre trial of the penumbra system enrolled 125 patients presenting within two hours of stroke.^27^ The primary endpoints were vessel revascularisation and device safety. Recanalisation rates were 81.6% and serious adverse rate was 3.2%. An NIHSS score showed improvement of > 4 points in 57.8% of patients and an mRS ≤ 2 at 90 days in 25% of patients. Mortality rates at 30 and 90 days were 26 and 32.8%, respectively. More recent studies with the device reported recanalisation rates of between 85 and 93%.^24^

A review of all studies to date with neurothrombectomy devices revealed widely varying rates of recanalisation (43–78% with MERCI retriever and 83–100% with the penumbra system). Rates of harm included symptomatic (0–10% with MERCI and 0–11% with penumbra system) or asymptomatic (28–43 and 1–30%, respectively) intracranial haemorrhage. Vessel perforation or dissection (0–7 and 0–5%, respectively) also varied by device.^28^ Predictors of poor outcome were age, history of stroke, and higher baseline severity scores. Successful recanalisation was the sole predictor of good outcomes.^28^

Despite the FDA approving the MERCI device in 2004 and the penumbra system in 2007 for use in acute ischaemic strokes, their clinical efficacy is yet to be fully established in a controlled outcomes trial.^24^ The data as published are enticing and one is already seeing an increase in the use of these devices. The main problems remain patient selection, type and nature of stroke and clinical outcome. The studies have focused on recanalisation as the sole measure of outcome but, as in the IV r-TPA trials, it should really be clinical outcome.

## Endovascular angioplasty and stenting

Balloon angioplasty with or without stent placement, as is used in patients with acute myocardial infarction, has been used to recanalise cerebral arterial occlusions. Unlike cardiac arteries which have the firm muscular support of the myocardium, cerebral arteries are suspended in cerebrospinal fluid and are hence more prone to dissections and tears. Furthermore, the approach to cerebro-arterial occlusions is often tortuous, making navigation much more difficult. Reperfusion rates of approximately 80% with mortality of about 30% have been reported.^24^ However the AHA guidelines do not recommend this form of treatment.^12^

## Stent-based thrombectomy

Self-expanding stents for cerebral use have advantages over balloon angioplasty as they can be delivered to the target vessel with reduced barotrauma, thereby decreasing the risk of rupturing or dissecting the cerebral vessel.^29^ Moreover, they adapt much better to the shape and anatomy of the affected artery.

The solitaire AB stent is a self-expanding microstent. The device is deployed at the level of occlusion and the clot is enmeshed in the stent and then removed proximally. Studies have shown successful revascularisation in patients presenting within eight hours of AIS.^24^A recent study suggest that recanalisation rates > 85% can be achieved with the Solitaire stent in anterior large-vessel occlusions, thereby substantially increasing the rate of good outcome for these patients with an otherwise poor prognosis.^30^

The above endovascular approaches for treatment of stroke are viable but costly. They require a dedicated stroke interventionalist with a support team of angiography technicians and nurses. The equipment needed to carry out these procedures is also expensive. Careful selection of patients is imperative in order to achieve maximum benefit. In South Africa, interventionalists are few, and hence this poses a greater difficulty in achieving the ultimate goal of early reperfusion.

## Multimodal reperfusion therapy

Faster and more complete recanalisation should translate into better patient outcomes. To achieve this, the trend in acute coronary syndromes has been to use multiple pharmacological agents and, increasingly, percutaneous coronary intervention. However, currently available data do not provide conclusive evidence for either the safety or efficacy of combinations of medications to improve cerebral perfusion. Data with regard to the usefulness of mechanical devices to augment the effects of pharmacological thrombolysis to treat AIS are also limited.

## Prevention of recurrent stroke

## Antiplatelets

Aspirin is widely used for the prevention of recurrent stroke in patients with transient ischaemic attack (TIA) and ischaemic stroke of arterial origin because it is effective and inexpensive. Aspirin reduces recurrent strokes rather than limiting the neurological consequence of a stroke.^12^

Two large trials evaluated aspirin for the treatment of acute ischaemic stroke.^31^,^32^ The Chinese Acute Stroke trial (CAST) and the International Stroke trial (IST) randomised just over 40 000 patients with acute ischaemic stroke. In conjunction, these studies concluded that aspirin given within 48 hours of stroke produces a modest but definite benefit with about 10 fewer deaths or recurrent strokes per 1 000 in the first few weeks. Oral aspirin (150–300 mg loading dose) given within 48 hours after ischaemic stroke is recommended. However, aspirin or other antithrombotic therapy should not be initiated within 24 hours if thrombolytic therapy is given.^4^

Dipyridamole plus aspirin, or clopidogrel alone, is more superior to aspirin alone in secondary prevention of strokes and other vascular events and their overall safety profiles are similar.^33^,^34^ However, a considerable proportion of patients discontinue dipyridamole therapy because of headache,^35^ and clopidogrel is more expensive than aspirin. Clopidogrel in conjunction with aspirin is not more effective than clopidogrel alone in preventing ischaemic strokes and other vascular events. Furthermore, this combination also increases the risk of major bleeding.^36^

Although the AHA guidelines do not recommend clopidogrel or dipyramidole either alone or in conjunction with aspirin for the treatment of acute ischaemic stroke, a recent systematic review, which assessed the clinical effectiveness and cost effectiveness of the above three agents, used either alone or in combination, concluded that for patients with ischaemic stroke or TIA, modified-release dipyridamole + aspirin, followed by aspirin alone, followed by clopidogrel, appears to be a cost-effective approach to the prevention of future occlusive vascular events.^37^

Intravenous glycoprotein IIb/IIIa inhibitors such as abciximab are currently not recommended for use in acute ischaemic stroke until more research is available.^12^ Newer antithrombotic agents that have shown efficacy in acute coronary syndromes, such as thienopyridine and prasugrel, and the non-thienopyridine, ticagrelor (a reversible ADP receptor antagonist) may be promising potential treatments for acute TIA and ischaemic stroke.^28^

## Anticoagulants

Data suggest that early anticoagulation with heparin or the low-molecular weight heparins/danaparoid does not lower the risk of early recurrent strokes nor does it halt neurological worsening. It also increases the risk of bleeding in the brain or other parts of the body. Hence it is not recommended for use in acute ischaemic strokes and should certainly not be given within 24 hours of thrombolytic therapy.^12^

Warfarin is universally used as an antithrombotic therapy for patients with TIA or ischaemic strokes of cardiac origin. Warfarin has been shown to reduce the risk of recurrent stroke or systemic embolism by about 61% in atrial fibrillation (AF) patients with recent TIA or ischaemic stroke.^38^ However warfarin does increase the risk of major extracranial haemorrhage. According to the South African stroke guidelines, dose-adjusted warfarin is recommended for patients with cardio-embolic ischaemic stroke or TIA associated with intermittent or persistent atrial fibrillation.^4^

Newer oral anticoagulants such as dabigatran, apixabin and rivaroxaban have emerged and trials on these drugs in stroke prevention for patients in AF appear promising. Recently, three large phase III randomised, controlled trials, the Randomized Evaluation of Long-Term Anticoagulation Therapy (RE-LY) trial, the Rivaroxaban Once Daily Oral Direct Factor Xa Inhibition Compared With Vitamin K Antagonism for Prevention of Stroke and Embolism Trial in Atrial Fibrillation (ROCKET AF), and the Apixaban for Reduction of Stroke and Other Thromboembolic Events in Atrial Fibrillation (ARISTOTLE) trial have shown that dabigatran, rivaroxaban and apixabin are each more efficacious than warfarin for preventing strokes in patients with AF, with lower rates of intracranial bleeding.^39^-^41^ Other advantages of these anticoagulants over warfarin are that they can be used in fixed doses and regular monitoring of anticoagulation intensity is not necessary.^42^

The FDA has approved dabigatran and rivaroxaban for stroke reduction in people with non-valvular atrial fibrillation.^43^ FDA approval for apixabin is pending. Three studies have compared these three oral anticoagulants for stroke prevention in AF patients.^44^-^46^ Apixaban was shown to be as effective as dabigatran but rivaroxaban was less effective than dabigatran.^44^ Apixaban was associated with less major bleeding than dabigatran or rivaroxaban.^44^ Dabigatran is more cost effective than rivaroxaban in terms of acute care and long-term follow up costs, as well as accrual of quality-adjusted life years.^45^ Apixaban is associated with less major and gastrointestinal bleeding than dabigatran and rivaroxaban.^46^ Randomised trials comparing the three drugs are required to confirm these findings.

Apixabin was also directly compared to aspirin in the Apixaban Versus Acetylsalicylic Acid to prevent Strokes (AVERROES) trial and shown to be more effective in reducing strokes compared to aspirin in AF patients who have had previous strokes or TIAs and who are unsuitable for or unwilling to take a vitamin K agonist.^47^

## Treatment of dyslipidaemia

Dyslipidaemia is a major risk factor for coronary heart disease but its role in ischaemic stroke is not clear. It is however associated with atherosclerosis, which causes strokes.4 A meta-analysis of 90 000 patients suggested that larger low-density lipoprotein cholesterol (LDL-C) reductions better reduce the risk of stroke.^48^

In five placebo-controlled studies with more than 40 000 patients with coronary heart disease, HMG-CoA reductase inhibitors (statins) reduced the risk of stroke by 19–50%.^49^ Of all the statins, atorvastatin is the most favourable.^49^ Simvastatin reduced major vascular event by 20%. AHA guidelines recommend using a statin to reduce the risk of recurrent stroke in patients with evidence of atherosclerosis, an LDL-C level > 100 mg/dl, and those who are without known coronary heart disease.^50^ Maximum benefit is attained with a reduction of the LDL-C level by at least 50% or below 70 mg/dl.

Other medications used to treat dyslipidaemia include niacin, fibrates and cholesterol-absorption inhibitors. Niacin and gemfibrozil are recommended (class 2b) for use in patients with ischaemic stroke or TIA with low high-density lipoprotein cholesterol (HDL-C.) levels. Lipid-lowering therapy is associated with delayed cardiovascular events and prolonged survival in patients with homozygous familial hypercholesterolaemia.^51^

## Treatment of large-artery atherosclerosis

Carotid revascularisation by carotid endarterectomy (CEA) or carotid angioplasty and stenosis (CAS) has been well documented in research to prevent strokes, provided there is appropriate case selection with the risk–benefit ratio being favourable for the patient. Current AHA guidelines advocate CEA for severe ipsilateral carotid artery stenosis (70–99%) in patients with recent TIA or ischaemic strokes, if the peri-operative morbidity and mortality risk is less than 6%.^50^ CEA can be considered for moderate carotid stenosis (50–69%) depending on patient-specific factors such as age, gender and co-morbidities and again, if the peri-operative morbidity and mortality risk is less than 6%.

With stenosis less than 50%, CEA or CAS is not recommended. When the diameter of the lumen of the internal carotid artery is reduced by 70% on non-invasive imaging or 50% on catheter angiography, CAS can be considered as an alternative to CEA for symptomatic patients at average or low risk of complications associated with endovascular intervention.

## Conclusion

There are many different ways of treating AIS. However the evidence points to IV r-tPA as the most effective and at present the gold standard of AIS treatment. Despite this, recanalisation treatments as described are flourishing at a rapid rate and more emphasis and interest are being directed at these areas. Although vessel recanalisation is vital to increasing the possibility of significant tissue reperfusion, clinical trials need to emphasise functional outcomes rather than reperfusion/recanalisation rates to adequately assess success of these devices/techniques.

Our view is that until these treatments become proven in large-scale studies, a greater endeavour should be made in resource-limited settings to expand facilities to enable IV r-tPA treatment within the 4.5-hour period following onset of the stroke. The resources required are small with the main costs being a CT scan of the brain and the cost of r-tPA. This can easily be done in any emergency facility in any part of the world.

What is needed is public awareness, and campaigns of ‘stroke attack’ should be revisited, especially in the resource-limited context. Intensive public-awareness campaigns (television, radio, the internet, social networking, newspapers) about early recognition of stroke as well as the importance of time constraints for a favourable outcome should be devised.

Education of emergency medical personnel as well as staff of smaller medical facilities is also crucial in enabling faster referral to a unit where thrombolysis can be done. This approach at present will halt to some extent the stroke pandemic that we are facing. Public profiling of stroke will strongly assist in dealing with risk factors and implementation of preventative strategies.

A final point that needs to be made is that imaging modalities are being refined towards identifying with more accuracy patients who would fulfill the criteria for IV thrombolysis following ischaemic stroke. Currently multi-parametric MRI studies are gaining momentum in terms of identifying such patients.

Specifically, diffusion–perfusion mismatch, gradient echo, MRA (MR angiogram) and FLAIR (Fluid Attenuated Inversion Recovery) sequences on MRI are being used. These have the advantage of providing more detailed information of the ischaemic penumbra and the extent of infarction that cannot be determined on CT scanning techniques. This reduces the risks of intracranial haemorrhage following thrombolysis. The disadvantage is that patients who would have otherwise qualified by CT criteria are likely to be rejected on the MRI criteria. Further refinements in this area are likely to occur that will make thrombolysis more objective with better outcomes.
